# Shoulder Injury Related to Vaccine Administration

**DOI:** 10.31486/toj.21.0114

**Published:** 2022

**Authors:** Carson Flores, Walter S. Choate, Richard Tupler

**Affiliations:** ^1^ Department of Radiology, Ochsner Clinic Foundation, New Orleans, LA; ^2^ Department of Orthopedic Surgery, Ochsner Clinic Foundation, New Orleans, LA; ^3^ The University of Queensland Medical School, Ochsner Clinical School, New Orleans, LA

**Keywords:** *COVID-19 vaccines*, *influenza vaccines*, *injections–intramuscular*, *shoulder injuries*

## Abstract

**Background:** Shoulder injury related to vaccine administration (SIRVA) is a recognized complication and possible source of morbidity associated with incorrectly administered intramuscular deltoid vaccinations. As this site is commonly used for intramuscular injection, both clinicians and vaccine administrators should be familiar with SIRVA to minimize risk and monitor for its clinical presentation.

**Case Report:** A 49-year-old male presented with shoulder pain that began 1 day after intramuscular administration of an influenza vaccine and point tenderness near the site of injection. Magnetic resonance imaging of the shoulder demonstrated focal osseous edema in the humeral head related to suboptimal needle placement.

**Conclusion:** Based on the combination of history, physical examination findings, and imaging findings, the diagnosis of SIRVA was made with confidence in this clinical scenario.

## INTRODUCTION

The deltoid muscle is the most superficial muscle overlying a significant portion of the glenohumeral joint and one of the most common targets for intramuscular vaccinations and other injections. Because of the deltoid muscle's commonality as a location for injections and its proximity to the underlying joint, the potential for complications from suboptimal vaccination technique is present. One such negative outcome is known as shoulder injury related to vaccine administration (SIRVA).

We present a case of SIRVA following an attempted intramuscular deltoid influenza vaccination with a classic set of clinical and radiographic findings.

## CASE REPORT

A 49-year-old male presented with a 3-month history of left shoulder pain. He reported that the pain began 1 day after receiving an influenza vaccination in his left shoulder. The patient received a 0.5 mL dose of injectable influenza, quadrivalent, preservative-free (IIV4; Fluzone Quadrivalent, Sanofi Pasteur), with the site of injection documented as within the left deltoid. A pharmacist at a large retail pharmacy administered the vaccine as part of the chain's Pharmacist Immunization Program.

The patient's initial pain was significant for several days, improved slightly without resolving, and then progressively worsened during the next 2 months, prompting presentation to the emergency department (ED). He reported the occasional “giving out” of his left arm when crawling on all fours, as was sometimes necessary in his occupation as a heating, ventilation, and air-conditioning technician. The pain was worst with overhead movements, quantified by the patient as 6 of 10 in severity, and negatively impacted his sleep. The patient denied any associated neck pain, shoulder swelling, or redness. The patient endorsed a history of chronic left shoulder pain with certain overhead activities that he had had for several years but that was tolerable; however, his presenting symptoms were different in quality and severity.

**Figure 1.**
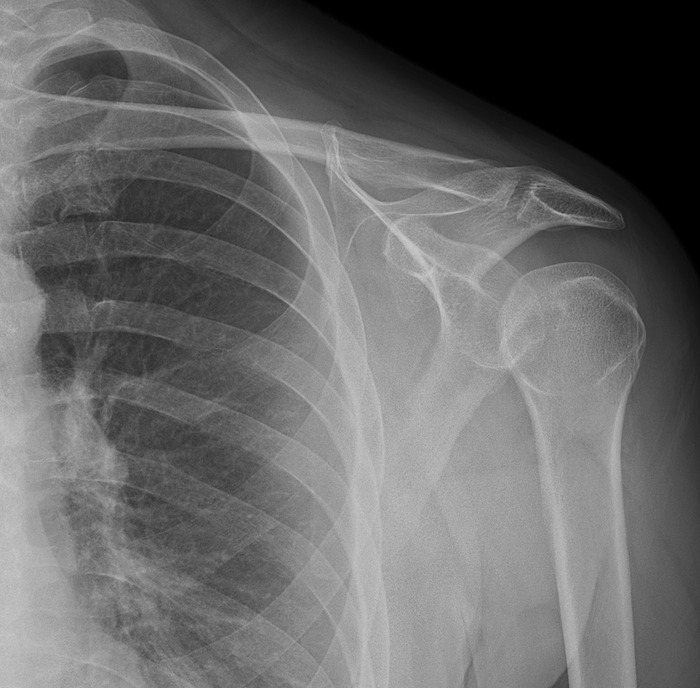
Conventional radiograph of the left shoulder demonstrated no acute pathology.

Physical examination findings on initial presentation were positive for severe point tenderness at the deltoid muscle, generalized tenderness about the left glenohumeral joint, and diffuse pain with cross-chest abduction. Range of motion was full. Radiographs of the left shoulder obtained in the ED demonstrated mild acromioclavicular joint osteoarthritis with otherwise no significant abnormality (Figure 1). The patient was discharged from the ED with prescriptions for naproxen 500 mg oral tablets and topical diclofenac-sodium 1% gel, as well as outpatient referral to orthopedic surgery.

**Figure 2.**
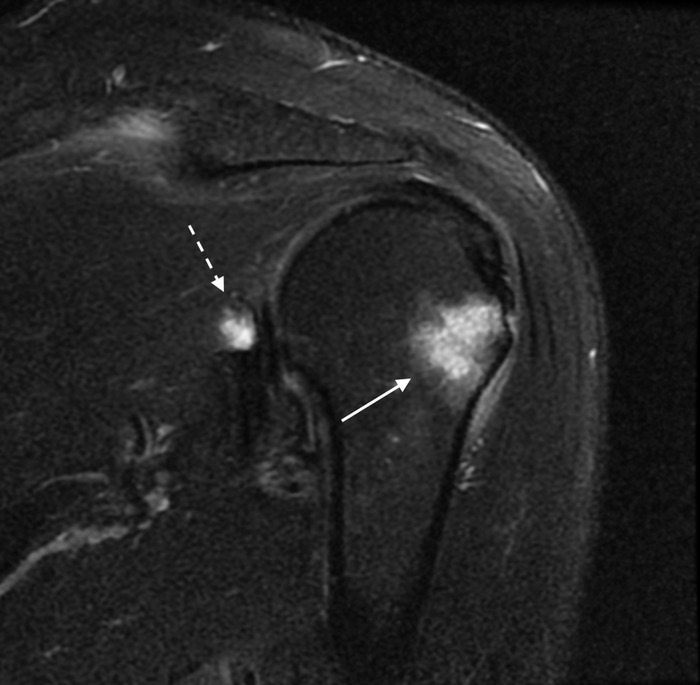
Coronal T2-weighted magnetic resonance imaging of the left shoulder with fat saturation shows focal osseous edema within the humerus at the level of the infraspinatus muscle insertion on the greater tubercle (arrow). A complex paralabral cyst can also be partially seen near the posterosuperior glenoid (dashed arrow).

**Figure 3.**
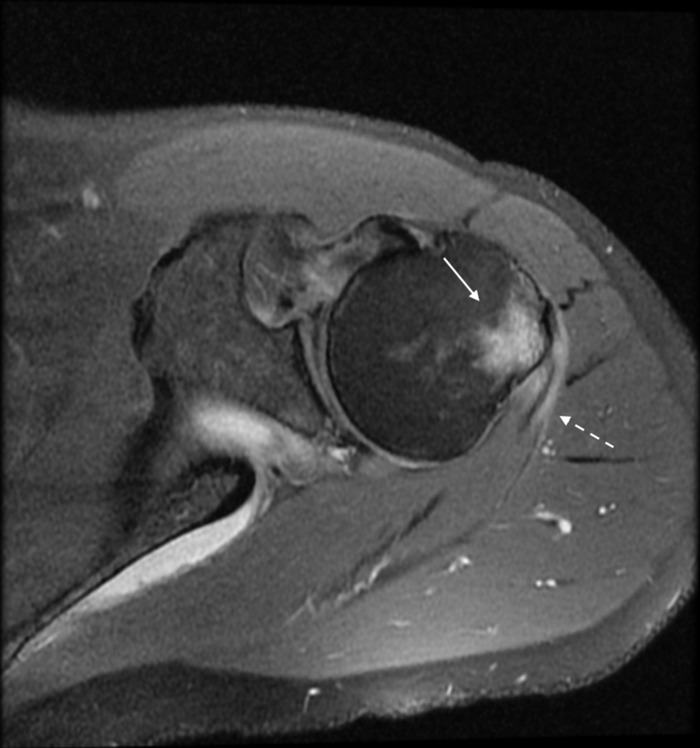
Axial proton density weighted magnetic resonance imaging of the left shoulder with fat saturation again demonstrates edema signal within the greater tubercle (arrow). Edema is also deep in the deltoid muscle and within the teres minor tendinous insertion (dashed arrow).

When the patient was seen in the orthopedic surgery clinic, he reported that the medications prescribed upon ED discharge had not improved his symptoms at all, and his symptoms were worse. He rated his pain as 8 of 10 in severity. Noncontrast magnetic resonance imaging (MRI) of the left shoulder demonstrated osseous edema within the posterolateral proximal humerus and humeral greater tuberosity at the level of the infraspinatus tendon insertion. Soft tissue edema was also seen within the teres minor tendinous insertion and subscapularis musculotendinous junction (Figures 2 and 3). Tearing of the posterosuperior and posterior glenoid labrum with a large paralabral cyst was noted.

The initial MRI report listed metastatic disease as a differential diagnosis for the osseous findings, so whole-body bone scan was recommended to evaluate for additional regions of abnormal bone marrow. Technetium-99m-methyl diphosphonate whole-body bone scan obtained approximately 2 weeks following the initial orthopedic surgery consultation was negative for any asymmetric or abnormal uptake suspicious for malignancy.

At a follow-up visit with orthopedic surgery following the shoulder MRI and bone scan, 4 months from the date of his influenza vaccination, the patient endorsed continued pain. Although he did not quantify the severity of the pain at this visit, he said it had continued to progress since the previous visit. The patient reported that his shoulder pain was never tolerable enough for him to participate in the physical therapy that was prescribed at the initial orthopedic surgery clinic visit.

SIRVA was diagnosed based on the onset of symptoms relative to the influenza vaccination and the imaging findings. The team of treating physicians felt that the patient's long-standing, less-severe shoulder pain was attributable to the labral tear seen on MRI and potentially related to overuse or repetitive motion, given the patient's profession and no apparent history of inciting trauma. A therapeutic injection of 40 mg triamcinolone acetonide was administered via posterior approach into the subacromial bursa with the intent of providing symptom relief.

Six months following the therapeutic shoulder injection, the patient reported no significant improvement in his symptoms that continued to negatively impact his daily life at work and when trying to sleep.

## DISCUSSION

While transient shoulder pain is an extremely common side effect of routine deltoid vaccine administration, pain persisting for longer than expected should prompt health care providers to investigate. SIRVA is a relatively rare although potentially underdiagnosed injury that results from improper vaccination technique. A retrospective analysis of data obtained from the US Food and Drug Administration and Centers for Disease Control and Prevention (CDC) co-sponsored Vaccine Adverse Event Reporting System (VAERS) between the years 2010-2017 identified 1,220 reports of atypical shoulder pain consistent with SIRVA, specifically following injection of inactivated influenza vaccine.^1^ Since 2017, when SIRVA was officially added as a covered injury to the National Vaccine Injury Compensation Program, a total of 424 more instances of SIRVA have been reported to VAERS.^2^ The majority of these cases were reported in 2021 (70.28%). A wide range of vaccines has been associated with SIRVA, including seasonal influenza, hepatitis A, varicella, pneumococcus, meningococcus, and both mRNA and viral vector forms of the coronavirus disease 2019 (COVID-19) vaccine. Case reports published in 2021 describe SIRVA occurring after COVID-19 vaccinations.^3-5^

The classic history of SIRVA is new onset of previously nonexistent shoulder pain less than 24 hours following vaccination or lack of expected improvement in the day following vaccination. The exact pathologic mechanism is unknown, but the prevailing theory is that an immune-mediated localized inflammatory response occurs in the setting of unintentional injection of antigenic material into the synovial soft tissues.^6^ A rare complication is progression to osteonecrosis of the humeral head.

Physical examination findings include pain with range of motion, numbness, weakness, and abnormal deep tendon reflexes not usually present. Common MRI findings include intrasubstance edema signal in deep muscular and/or tendinous structures, focal bone marrow signal abnormalities in the humeral head, and bursitis.^6,7^ Our case mirrors many of the clinical and radiographic features of typical SIRVA.

The CDC recommends that intramuscular injections be administered in the deltoid muscle with a 22- to 25-gauge needle in all adults aged ≥19 years. Additional guidance on needle length is provided based on patient body mass with the goal of administering the vaccine directly within the deltoid muscle rather than within the overlying subcutaneous fat or the underlying joint structures.^8^ As a rule of thumb, the recommended needle length for patients <70 kg, regardless of sex, is 1 inch. A 1.5-inch needle is more appropriate for men >118 kg and women >90 kg. Patients whose weight is between these parameters must be assessed by the vaccinator on a case-by-case basis for determination of the appropriate needle length.^8,9^

Vaccinators should have a basic familiarity with the anatomy of the injection area. The deltoid muscle is a large triangle-shaped muscle that overlies the shoulder. CDC vaccinator resources recommend using anatomic landmarks to determine an appropriate site of injection. First, locate the acromion process of the scapula, a bony landmark that extends over the lateral aspect of the shoulder joint. An optimal target is approximately 2 inches below the acromion and above the axillary fold/armpit. Injections are administered at a 90° angle to the skin.^8,10^

Direct intramuscular injection is desirable because optimal immunogenicity of the vaccine can be achieved thanks to the highly vascular nature of skeletal muscle relative to the surrounding fatty tissues, allowing prompt transmission of the antigen to the systemic circulation, quicker processing by the immune system, and reduced antigen enzyme denaturation.^11^ In unusual situations when intramuscular vaccination of the deltoid is not practical or feasible, the anterolateral aspect of the thigh or buttocks is an alternative site for potential injection. A study comparing hepatitis B vaccination immune response demonstrated statistically significant reduced antibody titers in patients who received vaccines in the buttocks, where more layers of fat are typically present, compared with shoulder-administered vaccines.^12^

Vaccines have traditionally been administered by a wide variety of health care workers, including nurses, nurse practitioners, physician assistants, doctors, emergency medical technicians, and paramedics. However, almost one-third of adult influenza vaccines are administered by pharmacists.^13^ To prepare for the national vaccination effort during the COVID-19 pandemic, on October 20, 2020, the US Department of Health and Human Services issued new guidance as part of the Public Readiness and Emergency Preparedness Act allowing qualified pharmacy technicians and state-authorized pharmacy interns to be covered under the scope of the act's protections.^14^ Prior to this act, pharmacy technicians had only been allowed to administer vaccines under certain circumstances in 3 states, although in a nationwide survey distributed online to community pharmacy technicians in 2018, 47.1% of pharmacy technicians responded that they were “unwilling” to administer vaccines.^15^ The urgent need for more vaccinators to meet the demand for COVID-19 vaccines has created a shift in attitudes among this group. Pharmacy technicians can now complete an accredited training course with hands-on training, developed in response to the pandemic, through the National Pharmacy Technician Association and become nationally certified for vaccine administration.^16^ Since October 2020, thousands of retail pharmacy technicians have been hired and trained in vaccine administration as this important group of health care workers has stepped up to fill this role.

## CONCLUSION

As the COVID-19 pandemic vaccine effort evolves, those learning vaccine administration techniques for the first time must be properly educated to avoid potentially debilitating complications such as SIRVA. We present this case as an advisory of the importance of proper technique and as a reminder of SIRVA for clinicians who may encounter similar patient presentations.

## Notes

*This article meets the Accreditation Council for Graduate Medical Education and the American Board of Medical Specialties Maintenance of Certification competencies for Patient Care and Medical Knowledge.*
